# Differences between migrasome, a ‘new organelle’, and exosome

**DOI:** 10.1111/jcmm.17942

**Published:** 2023-09-04

**Authors:** Xuebing Xu, Tong Wu, Renjie Lin, Shengze Zhu, Jie Ji, Dandan Jin, Mengxiang Huang, Wenjie Zheng, Wenkai Ni, Feng Jiang, Shihai Xuan, Mingbing Xiao

**Affiliations:** ^1^ Department of Gastroenterology, Affiliated Hospital of Nantong University Medical School of Nantong University Nantong China; ^2^ Medical School of Nantong University oral medcine192 Nantong China; ^3^ Research Center of Clinical Medicine Affiliated Hospital of Nantong University Nantong China; ^4^ Department of Clinical Laboratory Affiliated Dongtai Hospital of Nantong University Dongtai China

**Keywords:** exosomes, extracellular vesicles, migracytosis, migrasomes, organelle

## Abstract

The migrasome is a new organelle discovered by Professor Yu Li in 2015. When cells migrate, the membranous organelles that appear at the end of the retraction fibres are migrasomes. With the migration of cells, the retraction fibres which connect migrasomes and cells finally break. The migrasomes detach from the cell and are released into the extracellular space or directly absorbed by the recipient cell. The cytoplasmic contents are first transported to the migrasome and then released from the cell through the migrasome. This release mechanism, which depends on cell migration, is named ‘migracytosis’. The main components of the migrasome are extracellular vesicles after they leave the cell, which are easy to remind people of the current hot topic of exosomes. Exosomes are extracellular vesicles wrapped by the lipid bimolecular layer. With extensive research, exosomes have solved many disease problems. This review summarizes the differences between migrasomes and exosomes in size, composition, property and function, extraction method and regulation mechanism for generation and release. At the same time, it also prospects for the current hotspot of migrasomes, hoping to provide literature support for further research on the generation and release mechanism of migrasomes and their clinical application in the future.

## 1 INTRODUCTION

As a new organelle produced during cell migration, the migrasome is dependent on cell migration. The migrasome can mediate the release of cell contents, thus conveying information molecules and participating in the early embryo growth of zebrafish. In addition, the migrasomes that detach from cells are absorbed by the recipient cells and perform an increasingly important role in cell‐to‐cell communication. As a membrane‐wrapped organelle, the core component of the migrasome is the vesicle structure.^1^, ^2^ Exosomes with the same vesicle structure have been deeply studied and analysed and have achieved remarkable achievements in immune response, information transmission, tumour diffusion and homeostasis and play an important role in clinical treatment.^3^ This also implies the delivery function of migrasomes in the fields of embryo growth and tumour diffusion in the future, as well as infinite potential in clinical treatment.

## 2 THE SIZES OF MIGRASOMES AND EXOSOMES ARE DIFFERENT

As a kind of extracellular vesicle, exosomes are mostly discoid vesicles with diameters ranging from 40 nm to 160 nm.^3^ The migrasomes are organelles with monolayer vesicles, most of which are elliptical with a diameter of 1–3 μm. They also contain many vesicles, ranging from less than 10 to more than 300. Under scanning electron microscopy, they look like pomegranate‐like structures.^1^

## 3 THE COMPOSITION OF MIGRASOMES AND EXOSOMES IS DIFFERENT

**FIGURE 1:**
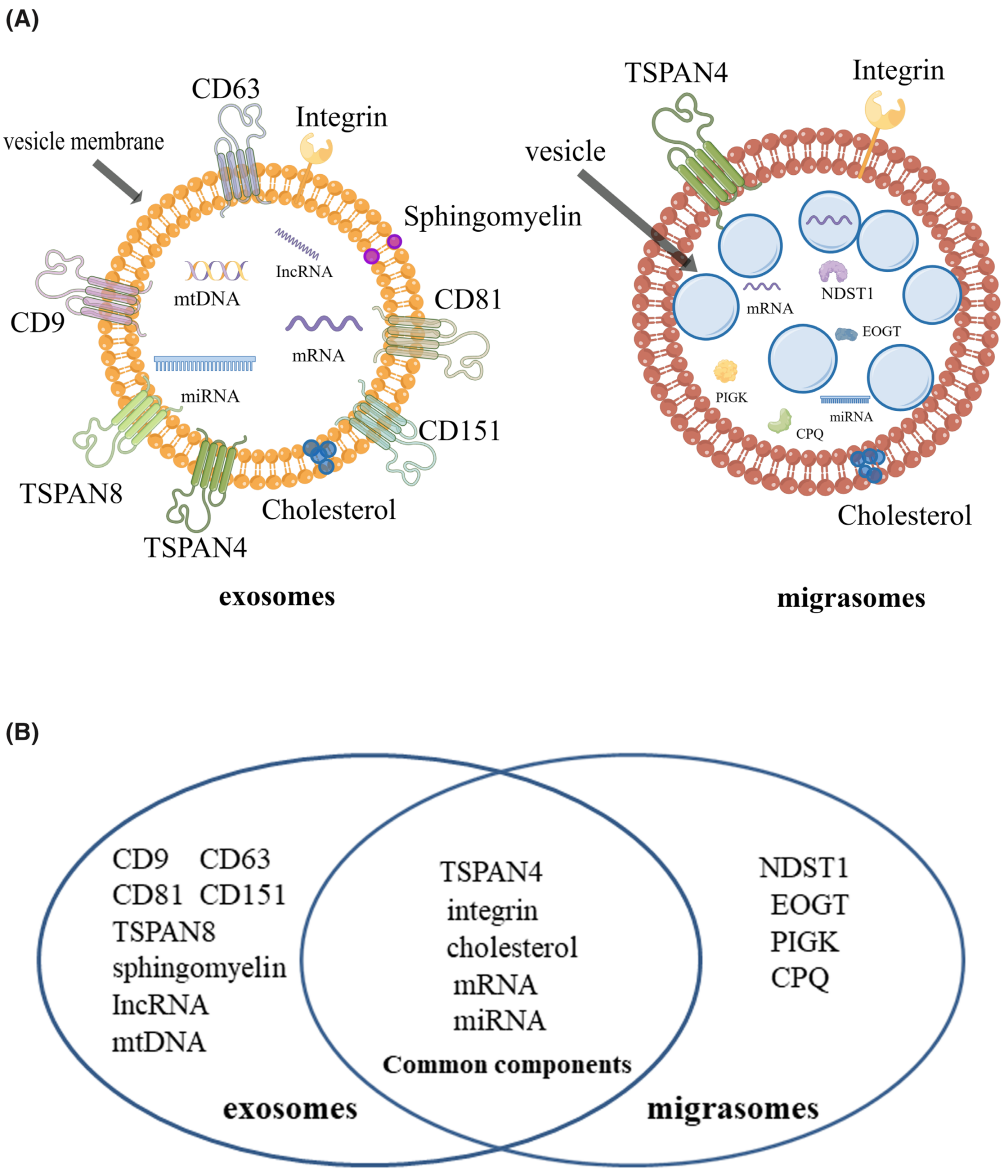
The composition of migrasomes and exosomes is different. (A) Composition of exosomes and migrasomes. (B) Differences and commonalities between exosomes and migrasomes in terms of components.

Exosomes consist of lipids, nucleic acids and proteins.^4^ There are many proteins on the vesicle membrane of exosomes, among which Tetraspanin 4 (TSPAN4) and integrin family adhesion molecules are the most abundant. CD9, CD63, CD81, CD151 and Tetraspanin 8 (TSPAN8), as adhesion molecules of the specific tetraspanin family, participate in cell migration, adhesion and other processes. There are many lipids in exosomes, including cholesterols and sphingomyelins. In addition, exosomes also contain many RNA and DNA molecules, such as mRNA, microRNA (miRNA), long noncoding RNA (lncRNA) and mitochondrial DNA (mtDNA) (Figure 1A).^3^, ^4^

The components of the migrasome include TSPAN4, cholesterol, integrin, mRNA and microRNA (miRNA).^5^, ^6^, ^7^, ^8^ There are many TSPAN4 and integrin on the migrasome. However, these two proteins also exist on exosomes, and it is difficult to determine the two structures just by detecting these proteins. Using mass spectrometry, the researchers found that these two structures shared only approximately 27% of the protein. Compared with exosomes, there are four specific proteins on the migrasome: N‐deacetylase and N‐sulfotransferase 1 (NDST1), EGF domain specific O‐linked N‐acetylglucosamine transferase (EOGT), phosphatidylinositol glycan anchor biosynthesis class K (PIGK) and carboxypeptidase Q (CPQ) (Figure 1A).^5^ Moreover, on migrasomes, the concentration of TSPAN4 is approximately four times higher than that of retraction fibres, and the concentration of cholesterol is approximately 40 times higher. Integrins are highly enriched in the migrasome but have low levels of retraction fibres. Due to the high concentrations of TSPAN4 and cholesterol, the rigidity of the membrane of the migrasome is greatly increased relative to the retraction fibres, and the membrane expands into the shape of a migrasome (Figure 1B).^9^, ^10^

## 4 THE PROPERTIES AND FUNCTIONS OF MIGRASOMES AND EXOSOMES ARE DIFFERENT

Exosomes are extracellular vesicles, while migrasomes are new organelles.^1^, ^4^

Exosomes are extracellular vesicles. The vesicle membrane of exosomes originates from the producing cells.^11^

The migrasome is a membrane‐wrapped cell structure that can be used by cells to release vesicles and other cell contents. In cell biology, organelles are defined as special subunits that perform specific functions in cells. Another key characteristic of an organelle is that it is generally wrapped within its own membrane. Therefore, on the basis of these criteria, we define the migrasome as a new organelle. The membrane structure of the migrasome is connected with the cell by retraction fibres. With the migration of the cell, the retraction fibres are gradually broken, and the migrasome is detached from the cell and released into the extracellular space. The migrasome spontaneously ruptures within a short time after being released into the extracellular space, then the smaller vesicles inside migrasome are also released into the extracellular space. The main components of the migrasome are extracellular vesicles after they detach from the cell, but most of the functions of the migrasome are completed before they detach from the cell. Cytoplasmic contents can be actively transferred to the migrasome, which means that the cell can use the migrasome to release cytoplasmic materials into the extracellular space. This is a new way to release cell contents and is the main reason that it is defined as an organelle rather than an extracellular vesicle.^1^ By contrast, exosomes perform almost all of their functions after they leave the cell.^3^

Almost all cells in the body are capable of releasing exosomes, so the function of exosomes depends on the type of cells from which they come.^12^ It can play a crucial part in the immune response, information transmission, tumour diffusion and maintenance of homeostasis.^3^ Though virtually all cells have the ability to release exosomes, the amount they release is not exactly the same, depending on the cell type and active degree, and the composition of exosomes largely reflects the composition of the protocell.^12^

**FIGURE 2:**
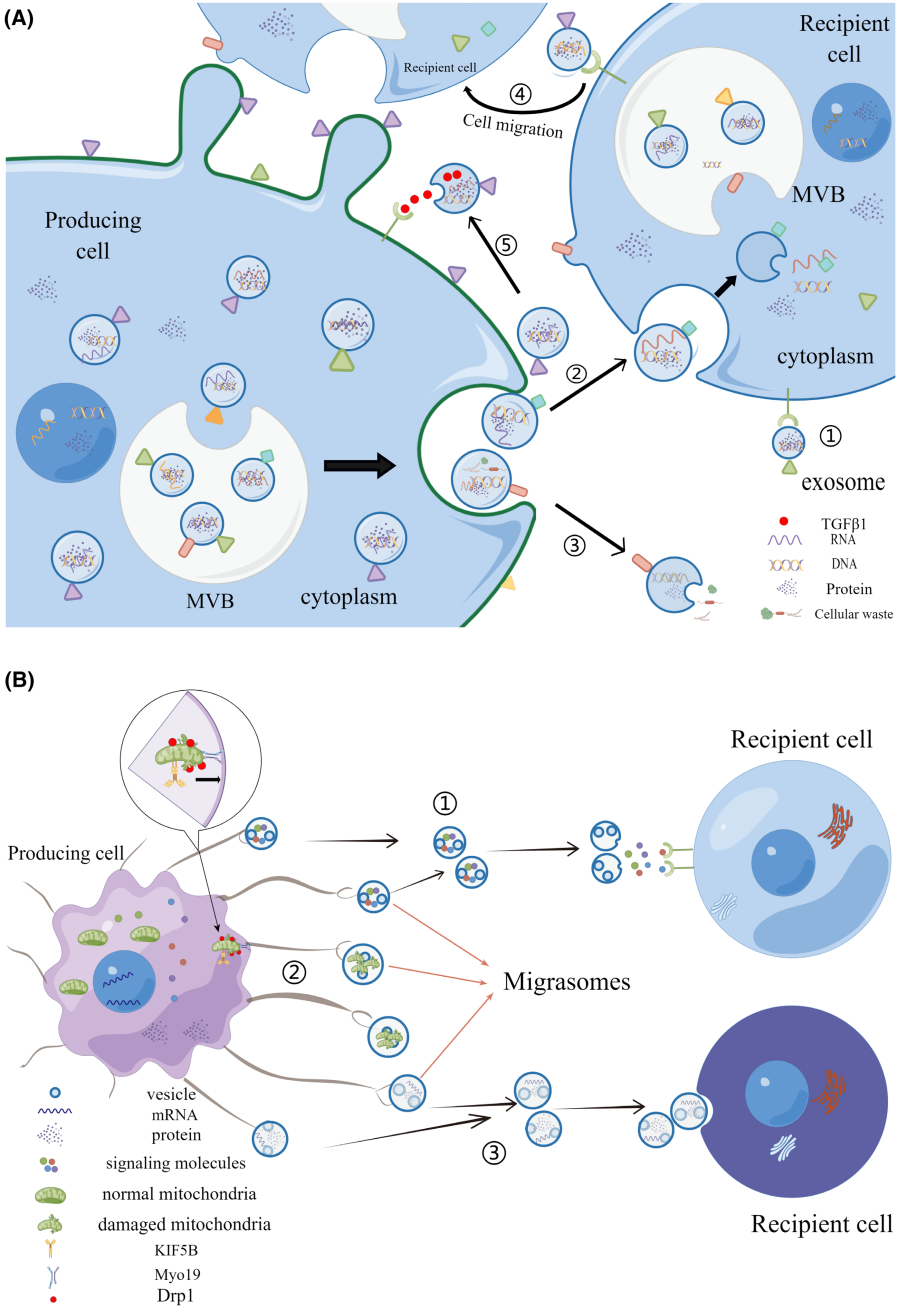
The functions of migrasomes and exosomes are different. (A) Five functions of exosomes: ① Exosomes act as ligands and directly bind to receptors on the surface of recipient cells. ② Exosomes can also only undertake the task of nucleic acids and proteins. ③ Exosomes transport waste products from cell damage to maintain a stable environment. ④ Exosomes can promote the generation of new cellular motion paths, such as cell migration. ⑤ Exosomes secreted by tumour cells contain factors that bind to their own receptors, such as TGFβ1, which can promote the growth of tumour cells. (B) Three functions of migrasomes: ① Migrasomes carry signalling molecules. When migrasomes leave the cells, they are lysed, and the signalling molecules are released and then bind to receptors on the surface of recipient cells. ② Migrasomes can dispose of damaged mitochondria. Damaged mitochondria are transported to the migrasomes. Three proteins, KIF5B, Drp1 and Myo19, are required for this process. KIF5B transports mitochondria to the plasma membrane, Myo19 tightly binds mitochondria to cortical actin, and Drp1 mediates final fission. ③ Migrasomes can mediate the transverse transfer of contents between cells, such as proteins and nucleic acids. Cell contents are first transported to the migrasomes and then released from the cell by the migrasome. Finally, cell contents and migrasomes are phagocytosed by recipient cells.

The functions of exosomes fall into five main categories. First, exosomes are capable of working as signalling molecules to directly stimulate recipient cells. Second, exosomes can only undertake the task of transporting proteins, nucleic acids and signalling molecules and do not participate in direct stimulation.^12^ Third, exosomes transport waste products from cell damage to maintain a stable cell environment.^13^ Fourth, exosomes can promote the generation of new cellular motion paths, such as cell migration and invasion, which are associated with the progression and deterioration of diseases, especially tumours.^3^ Fifth, exosomes secreted by tumour cells contain factors that bind to their own receptors, such as transforming growth factor beta 1 (TGFβ1), which can promote the growth of tumour cells (Figure 2A).^14^

The functions of exosomes show that exosomes own huge potential in clinical treatment in the future. They are mainly divided into three aspects. First, pathological cells, such as cancer cells, secrete exosomes containing a large number of bioactive factors conducive to the development of tumours, which can contribute to the invasion and spread of cancer cells. We can weaken the functions of exosomes by destroying their targets to delay disease progression.^15^, ^16^ Moreover, in view of the increasing number of cancer‐related factors that have been found in exosomes secreted by cancer cells, exosomes can be used as serum markers for partial cancer diagnosis.^17^ Second, for immune cells, exosomes can directly treat or slow disease progression. We can achieve better therapeutic effects by promoting the secretion of exosomes from immune cells.^18^ Third, along with the idea of secreting exosomes by immune cells, an increasing number of methods of drug delivery by exosomes have emerged, making full use of the transport capacity and targeting of exosomes to achieve the effect of personalized targeted therapy.^19^

The generation of the migrasome is inseparable from cell migration. According to the latest research progress, the function of the migrasome can be divided into three categories. First, the migrasome is rich in signalling molecules, such as cytokines, growth factors and chemokines. These signalling molecules can follow the migrasome to form a regional signalling center, and with the release of the migrasome, these signalling molecules can bind to the surrounding cell surface receptors and transmit relevant information. For example, it is involved in regulating the morphogenesis of embryonic organs in zebrafish. By experimenting with zebrafish embryos, researchers demonstrated that signalling molecules are enriched in the migrasome and can deliver complex information needed for embryonic growth and development within time and space limits.^2^, ^6^ Second, the migrasome can mediate a process of mitochondrial quality control called mitosis. During mitosis, mitochondria damaged by the stress response are transported to the migrasome, where they are then disposed of. Three proteins, kinesin family member 5B (KIF5B), dynamin‐related protein 1 (Drp1) and myosin 19 (Myo19), are required for this process. KIF5B transports mitochondria to the plasma membrane, Myo19 tightly binds mitochondria to cortical actin, and Drp1 mediates final fission and then transports mitochondria to the migrasome. In addition, the migrasome‐mediated mitochondrial quality control process can not only dispose of damaged mitochondria but also protect mitochondria from damage during the stress response.^20^ Third, the migrasome can mediate the transverse transfer of contents between cells. Cell contents are first transported to the migrasome and then released from the cell by the migrasome. Finally, cell contents are phagocytosed by surrounding cells, thus realizing transverse transfer between cells (Figure 2B).^6^

## 5 THE EXTRACTION METHODS FOR MIGRASOMES AND EXOSOMES ARE DIFFERENT

### 5.1 Extraction methods of exosomes

#### 5.1.1 Ultracentrifugation

Ultracentrifugation is the most generally used method to extract exosomes from samples.^21^ The theory of the method is to separate exosomes from the human sample based on the mass and sedimentation coefficient of exosomes. Briefly, the sample was centrifuged at different speeds. First, cell fragments and macromolecules were removed and centrifuged at 100,000 × g for 70 min to gain exosomes in the supernatant, and the temperature was controlled at 4°C.^22^ Exosomes can be extracted by ultrafast centrifugation, but they are time‐consuming, overly instrument‐dependent and have a low yield. Therefore, it is difficult to extract exosomes from small amounts of serum.^23^

#### 5.1.2 Density gradient centrifugation

**FIGURE 3:**
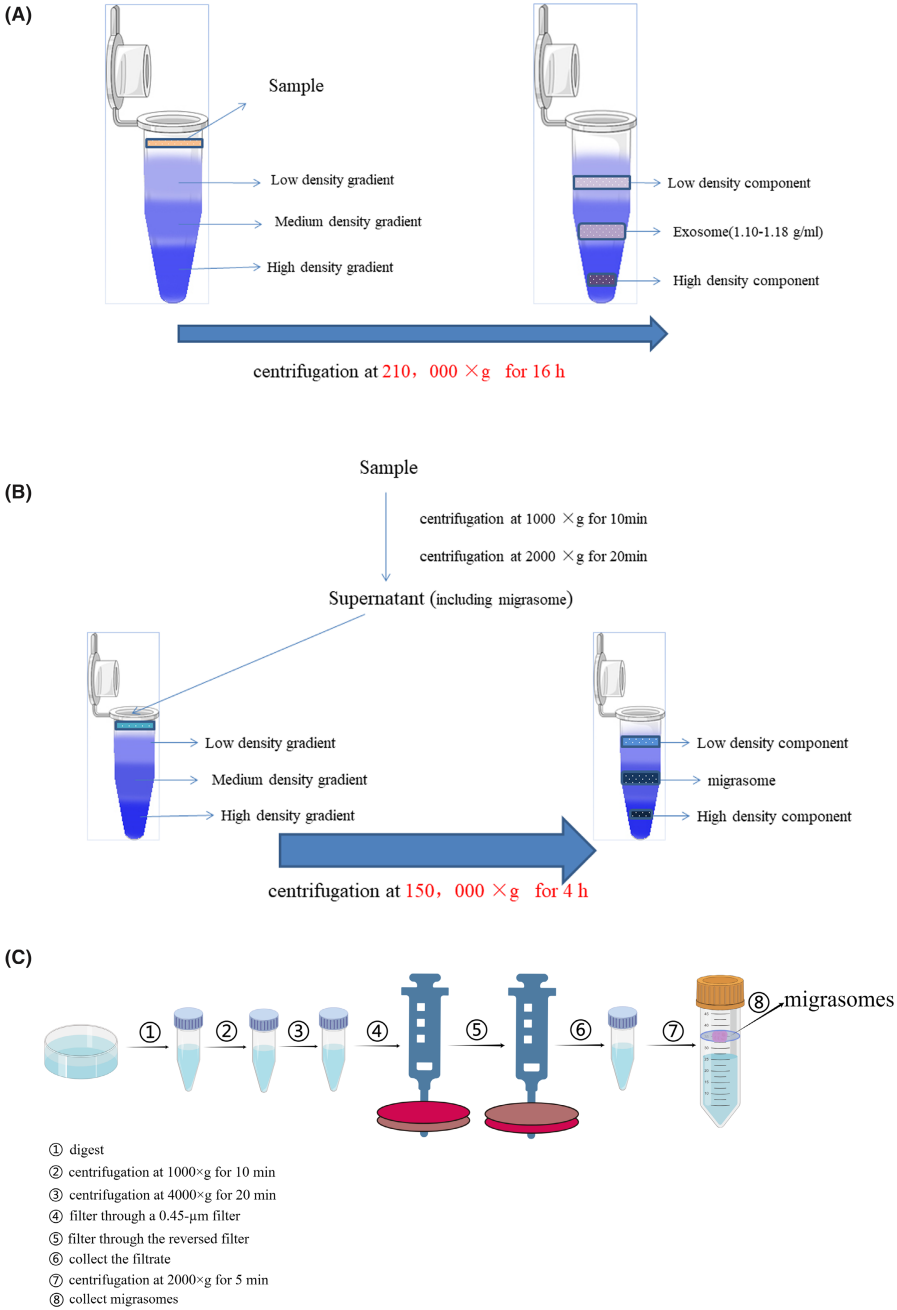
The extraction methods for migrasomes and exosomes. (A) Density gradient centrifugation for exosomes. (B) Density gradient centrifugation for migrasomes. (C) Isolation of migrasomes by centrifugation and filtration.

The principle of density gradient centrifugation is that different components in the sample will subside into their respective iso‐density regions under the effect of a certain centrifugal force so that exosomes can be separated from other components in the sample. We generally use sucrose as a gradient medium solution, set a sucrose gradient in the centrifuge tube, and then centrifuge at 210,000 × g for 16 h. The centrifugal temperature was controlled at 4°C.^24^ The concentration of exosomes in the density region (1.10–1.18 g/mL) was observed by sucrose density gradient centrifugation (Figure 3A).^25^ The advantage of this method is that exosomes are almost undamaged, and the separated components do not remix. The disadvantage is the long centrifugal time and low yield.^26^

#### 5.1.3 Ultrafiltration separation technology

The principle of ultrafiltration separation technology is that according to the pore size of the ultrafiltration membrane, certain relative molecular weight substances are allowed to pass through. The solvent and small molecules get through the ultrafiltration membrane, and the large molecules are trapped in the membrane. Therefore, the separation effect is achieved. The advantages are the short time required by this method and the high extraction amount of exosomes. The disadvantage is that substances of the same molecular weight cannot be distinguished, such as microbubbles and other extracellular vesicles, so the purity of the final product cannot be guaranteed.^27^

#### 5.1.4 Isolation of exosomes by the chip

An exosome complete isolation chip is designed to simplify exosome extraction. This method enriches and purifies exosomes from human samples through a nanopore membrane and then extracts intact exosomes ranging from 30 nm to 200 nm. Finally, concentrated exosomes were obtained from the filter membrane using a standard pipette. The advantages are that this method is simple and quick and can extract high‐yield exosomes from human samples, especially urine, for subsequent research.^28^

### 5.2 Extraction method of migrasomes

#### 5.2.1 Density gradient centrifugation

Sucrose density gradient centrifugation is generally used for cell isolation. Cells were collected and lysed in extraction buffer, and then the samples were centrifuged at 1000 × g for 10 min and 2000 × g for 20 min. At this time, the supernatant contained the original migrasome components, which were then placed in a sucrose density gradient and centrifuged at 150000 × g for 4 h. The enriched density gradient of the migrasome was observed and extracted by transmission electron microscopy (Figure 3B).^1^

#### 5.2.2 Isolation of migrasomes by centrifugation and filtration

The cells were washed twice with phosphate buffered saline (PBS), digested and centrifuged at 1000 × g for 10 min and then at 4000 × g for 20 min. After that, the supernatants were collected and saved, which were to be filtered through a 0.45‐μm filter. Afterwards a medical syringe containing 5‐mL PBS was used to squeeze the reversed filter. The filtrate was finally concentrated with a 100‐kDa ultrafiltration tube at 2000 × g and 4°C for 5 min to collect pellets which were migrasomes. (Figure 3C).^8^

## 6 THE REGULATORY MECHANISMS OF PRODUCTION AND RELEASE BETWEEN MIGRASOMES AND EXOSOMES ARE DIFFERENT

The first step of exosome formation is invagination of the plasma membrane, forming early endosomes. Over time, the membrane of the late endosome invaginates again, resulting in the formation of endosome containing intraluminal vesicles called multivesicular bodies (MVBs).^29^ The fusion of multivesicular bodies with the plasma membrane results in the release of intraluminal vesicles into the extracellular space as exosomes.^30^ The production and release of exosomes is a continuous process that can be divided into three main steps: the targeted delivery of MVBs, the junction of MVBs and plasma membrane and the fusion of MVBs and plasma membrane. This whole process works like an assembly line, transporting the multivesicular body (MVB) to its destination. However, the whole process requires the involvement of proteins on the surface of MVBs to ensure the accuracy of the whole process. It is mainly regulated by three proteins: SNARE proteins, RAB GTPase protein family and cytoskeletal proteins.^31^, ^32^, ^33^

Among them, the RAB GTPase family has different subcells.^34^ This allows them to play different roles in the delivery process, MVBs delivery, junction and fusion included.^31^ It is mainly RAB11, RAB27 and RAB35 of the RAB GTPase family. RAB27, mostly RAB27A, mediates the junction and fusion of MVBs to plasma membrane.^35^ RAB11 is involved in the delivery of MVB to plasma membrane.^36^ RAB35 mediates the junction of MVBs and plasma membrane.^37^

SNARE proteins control the junction and fusion of MVBs and plasma membrane. SNARE proteins include SNAP receptor proteins, which are located on the vesicle membrane (v‐SNARE), and SNAP receptor proteins, which are located on the target membrane (t‐SNARE). These two proteins determine the distinctive identification and fusion of vesicle membranes with target membranes.^32^

Cytoskeletal proteins offer power support during delivery and can drive MVB movement, where the kinesins drive forward movement and the dyneins drive negative movement.^33^, ^38^, ^39^

In addition to the regulation of proteins on the surface of MVBs, the microenvironment of cells can also induce the release of exosomes, and the exosomes secreted by cells take signalling molecules to alter the cell microenvironment. Meanwhile, changes in the cell microenvironment can promote the formation of exosomes to protect cells from damage as well.^40^, ^41^ For example, hypoxia in disease, increased glycolysis, an acidic extracellular environment, and increased intracellular calcium levels all promote the production and release of exosomes.^42^, ^43^, ^44^, ^45^

When a cell migrates, a membranous organelle that appears at the tip of its posterior contractile fibres is the migrasome. As the cell migrates, the contraction fibres connecting the migrasome and the migrant cell eventually break, and the migrasome detaches from the cell and is released into the extracellular space. In addition, the generation and release of migrasomes under molecular mechanisms have great reference significance.^1^

The principle of migrasome formation is that the membrane microdomains rich in TSPAN4 and cholesterol are split into micron‐scale macrodomains, which then gradually expand into migrasomes. Researchers have experimentally demonstrated that TSPAN4 and cholesterol are prerequisites for the formation of migrasomes. High concentrations of TSPAN4 and cholesterol are central factors in the formation of migrasomes, which greatly increase the rigidity of vesicles relative to retraction fibres, allowing the macrodomain to expand into the shape of the migrasome.

As the cells migrated, the researchers observed that the migrasomes stayed where they were formed, suggesting that something was holding the migrasomes in place of their formation. Using mass spectrometry, the researchers found that the migrasomes were rich in integrins and that integrins were gradually transferred from the contraction fibres to the migrasomes as the migrasomes formed. Thus, integrin formation seems to determine the location of the migrasome. It was further demonstrated that integrins and extracellular matrix (ECM) proteins are in an active ligand binding state and that integrins bind specifically to ECM proteins. The gene encoding integrin is knocked down, and the production of the migrasome is reduced. Thus, the pairing of integrins with ECM proteins determines the generation of the migrasome.^46^

The persistence and speed of cell migration also affect the generation of migrasomes. The researchers found that when cells migrate longer and faster, they produce more migrasomes, which are closely related to the retraction fibres on the cells.^47^

The long‐term absence of vimentin reduces the persistence and speed of cell migration and thus reduces the formation of migrasomes.^47^

## 7 CONCLUSIONS

In recent years, exosomes as a hot topic, have been widely studied and discussed, and the regulatory mechanism of their production and release is increasingly well known. Many findings of molecular regulation may help promote or reduce the release of exosomes, eventually leading to two kinds of pathogenic types for exosomes. First, diseased cells, such as tumour cells, release many exosomes, and exosomes they secrete contain numerous bioactive factors which are conducive to the progression of tumours and can contribute to the invasion and spread of cancer cells. Second, exosomes that immune cells secrete from the body are reduced, and exosomes secreted by immune cells can directly treat diseases or slow down the progression of diseases. Therefore, regulatory molecules are particularly important. We look forward to the discovery of more regulatory molecules and related regulatory mechanisms that can better regulate the production and release of exosomes and combine them with clinical treatment. Exosomes, as extracellular vesicles, originate from MVBs, so they can regulate the release of exosomes by regulating the delivery of MVBs to treat diseases.

Compared with exosomes, the role of the migrasome in disease is still in its infancy, and it is not clear whether it is the source of vesicles. Previous studies have proven that although the migrasome is a membrane‐wrapped vesicle structure similar to MVBs, it lacks lysosomal‐associated membrane protein 1 (LAMP1), the surface marker of MVBs. Therefore, it does not come from MVB. Moreover, the latest study found that as the cell migrates, when the retraction fibre ruptures, it forms a new extracellular vesicle. Similar to migrasomes, these small extracellular vesicles are dependent on cell migration and are called retractosomes. Mass spectrometry showed that the protein composition of the constrictor was highly similar to that of the migrasome but significantly different from that of the cell body.^48^ It is reasonable for us to hypothesize that the vesicle structure of the migrasomes and retractosomes is not derived from direct intracellular transport but is based on the migration of the cells and the function of the migrasomes. During the formation of migrasomes, the proteins in the migrasomes may partly originate from the proteins in the cells or new proteins produced in the process of regulating the formation of migrasomes. However, its specific mechanism remains to be further studied. Exosomes, as a hotspot, have been closely combined with clinical research, covering all organs of the body and solving many diseases. Although there are many differences between migrasomes and exosomes, commonalities can be found in the research ideas of exosomes, which can be used for reference and lay a foundation for the clinical treatment of migrasomes in the future.

## AUTHOR CONTRIBUTIONS

**Xuebing Xu:** Conceptualization (supporting); writing – original draft (lead); writing – review and editing (equal). **Tong Wu:** Formal analysis (equal); investigation (equal). **Renjie Lin:** Validation (equal); visualization (equal). **Shengze Zhu:** Investigation (equal); methodology (equal). **Jie Ji:** Project administration (equal); resources (equal). **Dandan Jin:** Resources (equal); supervision (equal). **Mengxiang Huang:** Methodology (equal); validation (equal). **Wenjie Zheng:** Formal analysis (equal); visualization (equal). **Wenkai Ni:** Investigation (equal); methodology (equal). **Feng Jiang:** Visualization (equal); writing – review and editing (equal). **Shihai Xuan:** Resources (equal); writing – review and editing (equal). **Mingbing Xiao:** Conceptualization (lead); writing – review and editing (equal).

## FUNDING INFORMATION

This study was supported by grants from the National Natural Science Foundation of China (grant number 82272624), the Natural Science Foundation of Jiangsu Province (grant number BK20211105), the Key Research and Development Plan of Jiangsu Province (grant number BE2019692, BE2020668), The Health Project of Jiangsu Province (grant number H2019072) and the Social Development Foundation of Nantong City (grant number JC22022001).

## CONFLICT OF INTEREST STATEMENT

The authors declare that they have no competing interests.

## Data Availability

Data sharing is not applicable to this article as no new data were created or analyzed in this study.
